# Oral Lichenoid Reactions Caused by Mesalazine for Treatment of Ulcerative Colitis: A Case Report and Review of the Literature

**DOI:** 10.1155/crid/4780630

**Published:** 2026-05-14

**Authors:** Smaragda Diamanti, Maria Myrto Solomou

**Affiliations:** ^1^ Department of Oral Medicine & Pathology, European University Cyprus School of Dentistry, Nicosia, Cyprus

**Keywords:** 5-aminosalicylic acid, case report, dentistry, fixed drug eruption, mesalazine, oral medicine

## Abstract

**Background:**

Mesalazine, also known as 5‐aminosalicylic acid (5‐ASA), is commonly used to treat ulcerative colitis and Crohn′s disease. While various adverse effects of mesalazine have been reported, oral lichenoid reactions (OLRs) are rarely associated with its use.

**Objective:**

The aim of this study is to present a rare case of mesalazine‐induced OLR and a brief review of the relevant literature.

**Case Presentation:**

A 45‐year‐old female with ulcerative colitis, treated with mesalazine for 2 years, presented with persistent roughness and burning sensation on the bilateral buccal mucosa for 6 months. Clinical examination revealed well‐demarcated, bilateral white striations. Patient refused any type of biopsy, but after ruling out other potential causes, mesalazine was discontinued, resulting in complete symptom resolution and near‐total disappearance of oral lesions within 1 month, suggesting the diagnosis.

**Methods:**

A literature review was conducted using PubMed, Scopus, and Cochrane Library databases to identify similar cases and relevant information on mesalazine‐induced OLRs.

**Results:**

This case represents one of the few documented instances of mesalazine‐induced lichenoid reactions and a rare report of such involvement exclusively in the oral cavity, distinguishing it from previously reported cases, which involved concurrent cutaneous lesions.

**Conclusion:**

This report highlights the importance of considering mesalazine as a potential etiological factor in patients presenting with oral lichenoid lesions, particularly those with inflammatory bowel disease. It underscores the need for increased awareness among clinicians to facilitate the timely recognition and management of similar cases.

## 1. Introduction

Mesalazine, also referred to as 5‐aminosalicylic acid (5‐ASA) or mesalamine, is an anti‐inflammatory agent widely used in the management of ulcerative colitis and Crohn′s disease. Its primary role is to reduce the frequency and severity of flares in patients with moderate to severe disease [1, 2]. Common adverse effects include acute reactions such as headache, nausea, and fever, while chronic or more severe complications like nephritis and pancreatitis are less frequent [3]. As the active component of sulfasalazine, mesalazine differs structurally and metabolically from its prodrug counterpart, which consists of mesalazine linked to a sulfapyridine moiety [3]. These differences contribute to distinct adverse effect profiles between the two medications [3]. While sulfasalazine‐induced lichenoid reactions are often attributed to the sulfapyridine moiety or excipients, reactions specifically driven by the 5‐ASA (mesalazine) component are distinct and less commonly reported [3]. Among the adverse effects associated with mesalazine, interstitial nephritis is uniquely attributed to its use, while pancreatitis has been reported to occur at a rate seven times higher compared to sulfasalazine [3]. In contrast, sulfasalazine is more frequently associated with blood dyscrasias, particularly in patients with rheumatoid arthritis, and a similar trend is observed for hepatic disorders [3]. Mesalazine has also been linked to various cutaneous adverse reactions, including nonspecific rashes, erythroderma, acute generalized exanthematous pustulosis, toxic epidermal necrolysis, and Stevens–Johnson syndrome during ulcerative colitis treatment [4–8].

Lichenoid hypersensitivity reactions (LHRs), often triggered by certain medications, are challenging to differentiate clinically and histologically from idiopathic lichen planus (LP) [9, 10]. The pathogenesis of LHRs is believed to be a T‐cell‐mediated Type IV hypersensitivity reaction. In this process, the drug or its metabolite binds to proteins in the basal keratinocytes or the basement membrane zone, creating a hapten–peptide complex. This complex is recognized as foreign by Langerhans cells, which then present the antigen to T‐lymphocytes. The subsequent activation of CD8^+^ cytotoxic T‐cells leads to the apoptosis of basal keratinocytes, resulting in the characteristic lichenoid tissue damage. Specifically for aminosalicylates like sulfasalazine and mesalazine, it has been hypothesized that active thiol groups in their chemical structure may facilitate this hapten formation, triggering the immune response [11].

Fixed drug eruptions (FDEs) in the oral cavity are rare, presenting as recurring lesions at the same site with each exposure to the offending medication. These lesions can vary in appearance, ranging from bullous and erosive to hyperpigmented, pruritic, or erythematous, and may involve the skin or genital mucosa in addition to oral sites [10].

This report presents a rare case of an oral lichenoid reaction (OLR) induced by mesalazine in a patient undergoing treatment for ulcerative colitis. To our knowledge, this is among the very few documented cases of mesalazine‐induced lichenoid reactions and the first to specifically describe such a reaction occurring exclusively in the oral cavity.

## 2. Methods—Literature Search Strategy

A comprehensive literature search was conducted using PubMed, Scopus, and the Cochrane Library databases up to July 2025. The search strategy utilized the following keywords and Boolean operators: (mesalazine OR mesalamine OR 5‐aminosalicylic acid OR 5‐ASA) AND (oral lichenoid reaction OR oral lichenoid lesion OR lichenoid hypersensitivity reactions OR fixed drug eruptions).

The search was not restricted by language or publication year. Inclusion criteria comprised systematic reviews, case reports, case series, and clinical studies documenting lichenoid reactions attributed specifically to mesalazine. Gray literature and case report databases were also screened to ensure comprehensive coverage. The initial search yielded eight results. After removing duplicates and screening titles/abstracts for relevance, two articles were selected for full‐text review.

## 3. Case Report

### 3.1. Diagnosis and Etiology

A 45‐year‐old female patient of Asian descent presented to a private oral medicine clinic in July 2020 with complaints of persistent roughness and a burning sensation on the bilateral buccal mucosa for the preceding 6 months. Upon detailed history taking, the patient reported a diagnosis of ulcerative colitis, managed with mesalazine (800 mg twice per day) for the past 2 years. She denied any additional medical conditions, concurrent medication use, a family history of LP, prior hepatitis B vaccination, or significant exposure to cinnamon‐containing products or other known allergens. An intraoral examination was performed using standard clinical protocols and documented with a digital camera (Canon EOS 2000D with a macro lens Canon EF 100 mm 1:2.8). Furthermore, there was no clinical or historical evidence of adjacent dental restorations, cutaneous involvement, or systemic manifestations suggestive of a broader disease process.

#### 3.1.1. Examination

**Figure 1:**
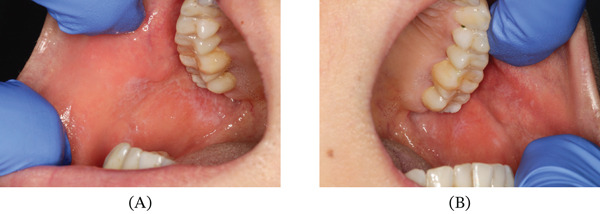
Intraoral photographs taken under standard clinical lighting at the initial presentation. (A) Right buccal mucosa and (B) left buccal mucosa showing characteristic lichenoid features, specifically extensive, well‐demarcated, white reticular striations (Wickham striae) appearing as lace‐like lines against an erythematous background.

Intraoral examination revealed well‐demarcated, bilateral white striations on the buccal mucosa (Figure 1).

Routine laboratory investigations, including complete blood count (CBC) and biochemistry, were performed and revealed no significant abnormalities, further excluding systemic causes.

#### 3.1.2. Treatment Objectives

The primary treatment objectives were to alleviate the patient′s reported burning sensation, eliminate mucosal roughness, and achieve clinical resolution of the lichenoid lesions. A secondary objective was to identify and manage the causative agent to prevent recurrence while ensuring the patient′s ulcerative colitis remained under control.

### 3.2. Differential Diagnosis

Based on the clinical presentation, the primary differential diagnoses included oral lichen planus (OLP) and drug‐induced oral mucosal lichenoid reaction (OMLR). Other conditions were considered and ruled out. Contact hypersensitivity (e.g., to dental restorative materials or cinnamon) was excluded due to the absence of recent dental work or dietary changes. Systemic conditions such as lupus erythematosus and graft‐versus‐host disease were ruled out based on the patient′s medical history and lack of systemic symptoms. Additionally, an FDE was deemed unlikely as the lesions presented with a reticular, striated pattern rather than the characteristic round or oval, hyperpigmented patches often seen in FDEs, and the lesions did not recur in the exact same location prior to this persistent presentation. Oral manifestations of the underlying ulcerative colitis itself (extraintestinal manifestations) were also considered in the differential diagnosis but were deemed less likely due to the lesion morphology and response to drug withdrawal. A biopsy to confirm the diagnosis was recommended, but the patient declined the procedure.

#### 3.2.1. Treatment Progress

Initial management consisted of prescribing a topical corticosteroid mouth rinse (prednisolone oral solution, 10 mg/mL, 2 mL twice daily). Despite adherence to therapy, there was no clinical improvement at the 2‐week follow‐up, which heightened the clinical suspicion of a medication‐induced lichenoid reaction. In collaboration with the patient′s gastroenterologist, mesalazine therapy was discontinued.

#### 3.2.2. Treatment Results

**Figure 2:**
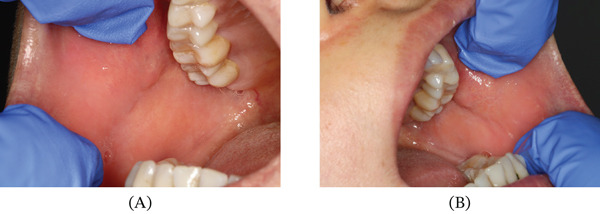
Intraoral photographs taken at the 1‐month follow‐up visit after the discontinuation of mesalazine. (A) Right buccal mucosa showing complete remission of the white striated lesions and return to normal mucosal color. (B) Left buccal mucosa showing significant improvement with near‐total disappearance of the lichenoid reaction, confirming the diagnosis.

At the 1‐month follow‐up, the patient reported complete resolution of symptoms, with near‐total disappearance of the oral lesions upon clinical examination (Figure 2).

These findings supported the diagnosis of an oral lichenoid drug eruption (LDE) secondary to mesalazine use. During the last follow‐up, the patient remained in remission without any new lesions for almost 1 year after the drug discontinuation.

A comprehensive timeline summarizing the clinical course, from the onset of ulcerative colitis to the resolution of oral lesions, is presented in Table 1.

**Table 1:** Timeline of the case.

Timepoint	Event
2 years prior to presentation	Patient diagnosed with ulcerative colitis; initiation of mesalazine.
6 months prior to presentation	Symptoms onset: The patient noticed roughness and a burning sensation on the bilateral buccal mucosa.
Initial visit	Clinical examination revealed bilateral white striations. Patient declined biopsy. Topical corticosteroids (prednisolone) were prescribed.
2‐week follow‐up	No clinical improvement despite adherence to topical therapy. Decision made to discontinue mesalazine in collaboration with the gastroenterologist.
1‐month postdiscontinuation	Follow‐up visit showing resolution of symptoms and near‐total disappearance of oral lesions. Diagnosis confirmed as mesalazine‐induced oral lichenoid reaction.

## 4. Discussion

LP is a chronic inflammatory condition that can affect the skin and various mucosal surfaces, including the oral, esophageal, and genital mucosa [12]. In the oral cavity, it commonly presents on the buccal mucosa, tongue, and gingiva, with clinical manifestations ranging from reticular white striations to confluent white and red plaques, ulcerations, or erosions. Although its exact cause remains unclear, it is believed to be an immune‐mediated response involving lymphocyte attacks on basal keratinocytes within the epithelium [12].

LDEs, considered a variant of LP, were first described in 1929 [13]. Drug‐induced oral lichenoid reactions (DIOLRs), now often categorized under oral lichenoid lesions (OLLs) or OLRs, mimic the clinical and histopathological features of idiopathic LP, and clinically distinguishing them can be challenging [13]. However, OLLs often exhibit a closer temporal association with the introduction of a causative agent, may present with a more diffuse or unilateral distribution, and can show deeper inflammatory infiltrates with eosinophils upon histopathological examination [13]. Lichenoid reactions can also exhibit a variable latency period [13]. In the case presented, the lesions appeared after 2 years of maintenance therapy, highlighting that such reactions can occur even after long‐term tolerance.

A wide range of medications has been implicated in OLRs, with varied presentations [13]. Aminosalicylates, such as mesalazine and sulfasalazine, are first‐line treatments for inflammatory bowel disease (IBD) [14]. Sulfasalazine is metabolized in the colon into sulfapyridine and mesalazine, with the two drugs exhibiting different adverse effect profiles [14]. OLRs attributed specifically to mesalazine are rarely reported. Alstead et al. [15] described two cases where oral and cutaneous lichenoid lesions developed during sulfasalazine therapy, with lesions recurring upon switching to mesalazine, suggesting that the aminosalicylic acid component may be the causative factor [15].

Reports directly linking mesalazine to OLRs remain sparse. While this suggests mesalazine‐induced OLRs are uncommon, it may also reflect underreporting or diagnostic challenges. To our knowledge, no prior reports have documented OLRs caused exclusively by mesalazine. While mesalazine‐induced lichenoid reactions involving the skin are documented, reports of involvement limited solely to the oral mucosa are exceptional. To our knowledge, this is the first reported case of a mesalazine‐induced lichenoid reaction occurring exclusively in the oral cavity without concurrent cutaneous lesions.

Diagnosing adverse drug reactions (ADRs) can be particularly difficult when invasive procedures like biopsies are declined by patients or are medically contraindicated. In the present case, the lack of histopathological confirmation is a limitation. However, a biopsy is not always mandatory when the clinical presentation is characteristic, and a clear temporal relationship with the drug exists. The diagnosis in this case was indicated by the “dechallenge” result, the complete clinical resolution of lesions within 1 month of discontinuing mesalazine. This resolution, combined with the exclusion of other local and systemic causes, supports the diagnosis of LDE despite the absence of histology. While diagnostic tools such as patch testing can be useful in distinguishing LDE from idiopathic LP, they were not pursued in this case. The rapid and complete clinical resolution following drug withdrawal provided sufficient diagnostic confirmation, rendering further testing unnecessary for patient management.

Tools such as the Naranjo algorithm, which assesses the probability of ADRs based on temporal association, alternative explanations, and prior case reports, help bridge this gap [16]. In this case, the Naranjo algorithm produced a score of 6, indicating a probable causal relationship between mesalazine and the observed OLR [16].

Other causality assessment tools, such as the WHO‐Uppsala Monitoring Centre (WHO‐UMC) system and the Liverpool Adverse Drug Reaction Causality Assessment Tool (LCAT), provide additional reliability for evaluating ADRs [17, 18]. Patch testing, which can distinguish OLRs from idiopathic OLP and identify causative allergens, is another valuable diagnostic tool, with diagnostic accuracy ranging from 16% to 68% depending on the study [19, 20].

This case underscores the importance of thorough clinical evaluation and the use of standardized diagnostic tools to support the identification and management of rare drug‐induced reactions.

It is also important to consider the potential pathophysiological link between OLRs and the patient′s underlying ulcerative colitis. Both conditions are fundamentally characterized by immune dysregulation and autoimmunity [21]. Ulcerative colitis is a chronic autoimmune disease, while lichenoid drug reactions are T‐cell‐mediated hypersensitivity processes that closely mimic autoimmune mechanisms [21]. This shared background of immune system hyperactivity suggests that patients with autoimmune disorders like ulcerative colitis may have an increased susceptibility to developing lichenoid reactions when exposed to offending agents [21].

While topical corticosteroids remain the standard first‐line therapy for symptomatic OLLs, recent literature has suggested exploring adjuvant therapies. Emerging evidence indicates that treatments such as ozone therapy and photobiomodulation could offer benefits in managing periodontal and mucosal conditions [22]. Additionally, the role of probiotics in modulating oral flora and potentially influencing mucosal immunity is an area of growing interest [23]. Future studies could investigate the efficacy of these noninvasive modalities specifically in the context of DIOLRs.

## 5. Limitations

A major limitation of this report is the absence of histopathological confirmation, as the patient declined a biopsy. Consequently, the proposed diagnosis is largely based on clinical exclusion criteria, the characteristic presentation, and the temporal relationship evaluated by the Naranjo algorithm (score of 6, *probable*). Furthermore, a drug rechallenge was not performed due to ethical and safety protocols to avoid reinducing severe symptoms. While the rapid and complete resolution of lesions upon withdrawal (positive dechallenge) strongly supports the diagnosis, it cannot provide absolute confirmation of the etiology without histological or allergological corroboration. Finally, patch testing was not utilized in this case; although it could have aided in distinguishing the reaction from idiopathic LP, the immediate clinical improvement following drug discontinuation guided the management decision.

## 6. Conclusion

This case highlights the necessity of considering mesalazine as a potential etiological factor in patients presenting with OLLs, particularly those with a history of IBD. It reinforces the importance of including mesalazine in the differential diagnosis of unexplained oral lesions. The complete resolution of symptoms and lesions upon discontinuation of mesalazine supports its role as the causative agent. This report adds to the limited body of literature on mesalazine‐induced OLRs and underscores the need for increased awareness among clinicians to facilitate timely recognition and management of similar cases.

## Author Contributions

All the authors have provided their intellectual contribution to the case report.

## Funding

No funding was received for this manuscript.

## Disclosure

The final draft was read and approved by each of the authors.

## Ethics Statement

Written informed consent was obtained from the patient for the publication of this case report and accompanying images.

## Conflicts of Interest

The authors declare no conflicts of interest.

## Data Availability

The data that support the findings of this study are available from the corresponding author upon reasonable request.
